# A retrospective cohort study of tamoxifen versus surgical treatment for ER-positive gynecomastia

**DOI:** 10.1186/s12902-023-01310-9

**Published:** 2023-03-13

**Authors:** Weili He, Weidong Wei, Qing Zhang, Rongzhao Lv, Shaohua Qu, Xin Huang, Juan Ma, Ping Zhang, Hening Zhai, Ningxia Wang

**Affiliations:** 1 Department of Breast Surgery, the First Affiliated Hospital of Jinan University, 510630 Guangzhou, China; 2 Department of Breast Surgery, Sun Yat-sen University Cancer Center, 510630 Guangzhou, China; 3 Department of Laboratory, the First Affiliated Hospital of Jinan University, 510630 Guangzhou, China; 4 Department of Ultrasonography, the First Affiliated Hospital of Jinan University, 510630 Guangzhou, China; 5 Department of Digestive Endoscopy c Center, the First/Fifth Affiliated Hospital of Jinan University, No. 613, Huangpu Avenue West, Tianhe District, 510630 Guangzhou, China

**Keywords:** Gynecomastia, Tamoxifen, Surgery, Recurrence, Complications

## Abstract

**Background:**

Gynecomastia is a common condition in clinical practice. The present study aimed to review the clinical data of ER-positive gynecomastia patients treated by tamoxifen (TAM) versus surgery and discussed the clinical effects of the two treatment strategies.

**Method:**

We retrospectively collected the clinical indicators of patients with unilateral or bilateral gynecomastia who received treatment at our hospital between April 2018 and December 2021. Depending on the treatment received, the patients were divided into TAM and surgery groups.

**Result:**

A total of 170 patients were recruited, including 91 patients in TAM group and 79 patients in surgery group. The age of the patients differed significantly between the TAM and surgery groups (P < 0.01). The estrogen level was closer in patients with stable and progressive disease, but significantly different in patients of glandular shrinkage in TAM group (P < 0.01). The proportion of patients achieving stable disease was higher among those with clinical grade 1–2. Among patients classified as clinical grade 3, the proportion of patients achieving glandular shrinkage of the breast was higher after TAM treatment (P < 0.05). The age and length of hospital stay were significantly different in patients undergoing open surgery than minimally invasive rotary cutting surgery and mammoscopic-assisted glandular resection (P < 0.01). Patients had significantly different complications including mild postoperative pain, hematoma, nipple necrosis, nipple paresthesias and effusions among the surgery subgroups (all P < 0.05). The estrogen level and the type of surgery were significantly different between the surgical recurrence and non-recurrence subgroups (P < 0.05). The difference in the thickness of glandular tissues upon the color Doppler ultrasound also reached a statistical significance between the two groups (P = 0.050). An elevated estrogen level was a factor leading to TAM failure. Among surgical patients, the thickness of glandular tissues, estrogen level, and type of surgery performed were risk factors for postoperative recurrence (all P < 0.05).

**Conclusion:**

Both treatment strategies can effectively treat gynecomastia, but different treatment methods can benefit different patients. TAM treatment is more beneficial than surgery for patients who cannot tolerate surgery, have a low estrogen level, and are clinical grade 1–2. Surgery treatment is better than TAM for patients of clinical grade 3. Different surgery options may lead to different complications. Patients with a greater glandular tissue thickness and a higher estrogen level were shown to have a higher risk of recurrence.

## Background

Gynecomastia is a common medical condition characterized by abnormal development of breast tissues and abnormal hyperplasia of breast connective tissues in men, which results from an estrogen and androgen imbalance due to physiologic or pathologic factors. Gynecomastia can occur unilaterally or bilaterally, typically presenting with abnormal enlargement of the breasts in men [1]. About 25% of boys and men have physiologic gynecomastia, especially teenage boys. Physiologic gynecomastia is usually benign [1–3]. Pathologic gynecomastia is usually caused by a hormonal imbalance, medications, endocrine diseases, chronic conditions, or systemic diseases [1, 4]. Estrogen level fluctuation or poor estrogen metabolism is an important factor affecting breast health in men [5]. Oral estrogen receptor antagonists, such as tamoxifen (TAM) and clomiphene, are common agents for gynecomastia treatment and can relieve breast pain and hyperplasia [6, 7]. Medications closely related to gynecomastia include spironolactone, human growth hormone (hGH), estrogen, human chorionic gonadotrophin (hCG), anti-androgens, and GnRH analogues [8]. These medications should be used carefully in the clinic for the sake of its close regulations of the human body. Chronic health conditions, including thyroid disorders, hypogonadism, and renal insufficiency, are also common causes of gynecomastia [1, 4]; however, if gynecomastia is secondary to other diseases, the underlying diseases will need treatment.

Consolation, continuous follow-up observation, and lifestyle changes (e.g., weight loss) are usually used to control gynecomastia [1, 4, 9]. Due to psychological and cosmetic considerations, medications and surgical treatment are becoming increasingly preferred among patients [1, 4, 9]. Several studies have recommended the use of TAM, an effective agent for gynecomastia [6, 10]. In one cohort study, TAM was shown to achieve complete remission in 90% of gynecomastia patients [11]. Another study recommended the use of TAM at an early stage of gynecomastia. Surgical intervention is the preferred option for gynecomastia patients with a course of disease longer than 12 months [12]. Leung et al. [13] recommended surgery for drug-resistant gynecomastia patients. Serretta et al. reported that TAM prevents bicalutamide-induced gynecomastia and breast pain [14]. To date, however, there have been few reports comparing the efficacy of TAM and surgical treatment for gynecomastia.

We determined the estrogen level in gynecomastia patients and compared the efficacy of TAM and surgical intervention. We also determined the expression of estrogen receptor (ER) in surgical patients and discussed the clinical treatment strategy for ER-positive gynecomastia patients.

## Materials and methods

### Study design

This study was approved by our institutional ethical review committee. We retrospectively collected the clinical data of 236 patients with unilateral or bilateral gynecomastia who received treatment at our hospital between April 2018 and December 2021. The patients chose the treatment strategies for gynecomastia after knowing detailed information on their clinical features and the two different treatments. Depending on the treatment the patient chose and received, the patients were divided into TAM and surgery groups. TAM was prescribed at a dose of 10 mg orally once daily for 3–6 months consecutively. These patients were followed up after discontinuation of TAM within 6 months to check whether there was recurrence. The following surgeries were performed: subcutaneous mastectomy with preservation of the nipple-areola complex via a peri-areolar incision; subcutaneous mastectomy by vacuum rotary cutting; and mammoscopic-assisted subcutaneous mastectomy with preservation of the nipple-areola complex. Data were collected of the following indicators: patient age; breast nodule grading based on the physical examination; results of color Doppler ultrasound; estrogen level; type of surgery performed; postoperative complications; and length of hospital stay. ER expression, a pathologic indicator, was determined. The patients receiving surgeries were followed up within 6 months after operations. During the follow-up period, the degree of patient satisfaction and recurrence were included for analysis. Recurrence was detected by color ultrasound during follow-up.

### Inclusion and exclusion criteria

The inclusion criteria were as follows: (1) The patients satisfied the diagnostic criteria for gynecomastia. Specifically, the patients had breast pain and discomfort. Breast nodules were palpable on physical examination with clear boundaries, a rough texture, and good mobility. The majority of breast nodules were located below or around the areola. There was tenderness pain, but no adhesions to the skin. The color Doppler ultrasound findings were consistent with gynecomastia. (2) The patients were > 18 years of age and willing to receive medications or strongly requested medications or surgical treatment. The patients signed the written consent form. (3) The patients had no severe heart, liver, kidney, and hematologic diseases, or any surgical contraindications. (4) The patients had no significant adverse reactions to oral TAM. (5) The patients received complete clinical and pathologic treatment. The exclusion criteria were as follows: confirmed cases of male breast cancer; no TAM or surgical treatment; intolerant to oral TAM; and surgical contraindications.

### Grading method for breast nodules

The glandular tissue thickness in the areola was determined by color Doppler ultrasound [15] and the maximum diameter along the direction of the vertical thickness was determined. The glandular tissue thickness based on color Doppler ultrasound is considered the gold standard, and gynecomastia was diagnosed if the glandular tissue thickness was ≥ 2 mm. Simon’s classification for the clinical grading of gynecomastia was used [16]. Depending on the degree of breast enlargement and skin redundancy, gynecomastia was divided into three grades: grade 1, minor breast enlargement with no skin redundancy; grade 2, moderate breast enlargement without skin redundancy; and grade 3, marked breast enlargement with skin redundancy.

By referring to the published literature [17], the TAM group was further divided into two sub-groups (patients achieving glandular shrinkage or a stable disease [collectively defined as responsive]; and patients with progressive disease [defined as unresponsive]). The surgical group was also divided into two sub-groups (patients without surgical recurrence [defined as responsive] and patients with surgical recurrence [defined as unresponsive]).

### ER detection

ER expression was determined using the EnVision method with ready-to-use ER (clone no.: EP1; Dako, Copenhagen, Denmark). The tissue sections were dewaxed and subjected to antigen retrieval (97 ℃ for 30 min). Then, the sections were washed in PBS 4 times for 3 min each time. The working solution of primary antibodies was added dropwise to incubate the sections for 60 min, followed by washing with PBS four times for 3 min each time. The sections were further incubated with FLEX/Mouse (LINKER; DAKO, Denmark), which was added dropwise for 15 min, followed by washing with PBS four times for 3 min each time. The sections were then incubated with FLEX/HRP (secondary antibody), which was added dropwise for 30 min. The sections were washed with PBS four times for 3 min each time. The incubation was continued for 3–5 min by adding 3,3’-diaminobenzidine (DAB; DAKO, Denmark) dropwise to the sections. The incubation duration varied with the staining intensity. After color development, the tissues were washed, soaked in hematoxylin for 5 min, then rinsed with running water. Differentiation was done using 1% hydrochloric acid-ethanol for 1–10 s. Next, the sections were washed with running water. After returning to blue for 4–5 s, the sections were washed with running water. Next, the counterstained sections were successively dehydrated in 70%, 85%, and 95% ethanol for 1 min. Finally, the tissue sections were dried and sealed with neutral balsam.

### Statistical analysis

All statistical analyses were performed using SPSS 18.0 software. For univariate analysis, the chi-square test with a four-fold table was used to analyze the correlation between the data. For multivariate analysis, multivariate logistic analysis was used to analyze the correlation between surgical recurrence and clinical indicators.

## Results

### Baseline indicators

A total of 236 male patients with gynecomastia were identified. According to the above inclusion and exclusion criteria, 66 patients who did not receive TAM or surgical treatment were excluded. Therefore, 170 patients were included. There were 91 patients in the TAM group and 79 patients in the surgery group. The following surgeries were performed: subcutaneous mastectomy with preservation of the nipple-areola complex via the peri-areolar incision (n = 36); subcutaneous mastectomy by vacuum rotary cutting (n = 39); and mammoscopic-assisted subcutaneous mastectomy with preservation of the nipple-areola complex (n = 4).

The comparison of baseline indicators between the two groups is shown in Table 1. Patients in the TAM group were older than patients in the surgical treatment group (average age: 36.40 vs. 28.85 years, P < 0.01). The proportion of patients with clinical grade 2 was significantly higher than patients with clinical grades 1 and 3 in both groups. Patients in the surgical treatment group were younger than patients in the TAM group. The proportion of patients with clinical grade 3 was significantly higher in the surgical treatment group than the TAM group (P = 0.043). The two groups of patients did not differ significantly with respect to glandular tissue thickness (91 mm vs. 79 mm) and estrogen level (37.60 vs. 40.99) (P > 0.05).

**Table 1:** Baseline indicators

Indicators		TAM group	Surgery group	P-value
Number		91	79	
Age(year)		36.40	28.85	0.000
clinical staging	Grade 1	7(7.7%)	8(10.1%)	0.043
	Grade 2	64(70.3%)	41(51.9%)	
	Grade 3	20(22.0%)	30(38.0%)	
Glandular tissue thickness(mm)		91	79	0.172
Estrogen level		37.60	40.99	0.351

### Correlation between the efficacy of TAM treatment and the clinical indicators

The correlation between the efficacy of TAM treatment and the clinical indicators was shown in Table 2. Ninety-one patients were treated with TAM, 21 of whom achieved glandular shrinkage, and 41 had stable disease and 29 had progressive disease based on efficacy evaluation. The choice of TAM treatment was not correlated with age or glandular tissue thickness based on color Doppler ultrasound findings; however, the glandular tissues were thicker in patients achieving glandular shrinkage after medication (P > 0.05). The estrogen levels were similar in patients with stable and progressive disease, but significantly different in patients of glandular shrinkage in TAM group (P < 0.01). The clinical grade was also correlated with the efficacy of TAM treatment. The proportion of patients achieving stable disease was higher among those with clinical grade 1-to-2. Among patients classified as clinical grade 3, the proportion of patients achieving glandular shrinkage of the breast was higher after TAM treatment (P < 0.049).

**Table 2:** The correlation between the efficacy of TAM treatment and the clinical indicators

Indicators		efficacy of TAM treatment			P-value	Χ^2^
		glandular shrinkage	stable	progressive		
Number		21	41	29	0.083	
Age(year)		29	41	21	0.358	
Glandular tissue thickness(mm)		9.11	7.41	7.96	0.063	
Estrogen level		46.74	34.51	31.00	0.001	
clinical staging	Grade 1	0	4	3		
	Grade 2	18	32	14	0.049	9.539
	Grade 3	11	5	4		

### Correlation between the type of surgery and clinical indicators

Seventy-nine patients completed surgical treatment, including 36 patients who underwent subcutaneous mastectomy with preservation of the nipple-areola complex via the peri-areolar incision, 39 patients underwent subcutaneous mastectomy by vacuum rotary cutting, and 4 patients underwent mammoscopic-assisted subcutaneous mastectomy with preservation of the nipple-areola complex. The results of correlation analyses between the indicator data are shown in Table 3. The patients undergoing open surgery were significantly older in age compared with the other two groups. The patients undergoing minimally invasive rotary cutting surgery and the mammoscopic-assisted glandular resection were comparable in age (P = 0.032). The patients undergoing open surgery had a longer hospital stay; however, there was no significant difference in the length of hospital stay between patients undergoing minimally invasive rotary cutting surgery and mammoscopic-assisted glandular resection (P < 0.01). The type of surgery was not significantly correlated with glandular tissue thickness based on color Doppler ultrasound, the estrogen level, ER expression, and clinical staging (P > 0.05). Mild pain was more common after minimally invasive procedures; however, a greater proportion of patients were suffering from moderate pain after open surgery (P < 0.01). Postoperative hematomas were more common after minimally invasive procedures (P = 0.040). Among patients undergoing open surgery, nipple necrosis (P = 0.01), nipple paresthesias (P = 0.07), and postoperative effusions were the most common complications (P = 0.02).

**Table 3:** The correlation between the type of surgery and clinical indicators

Indicators		Surgery types			P-value	Χ^2^
		open surgery	minimally invasive rotary cutting surgery	mammoscopic-assisted glandular resection		
Number		36	39	4	0.100	
Age(year)		31.72	26.77	23.25	0.032	
hospital stay		4.67	2.44	2.5	0.000	
Glandular tissue thickness(mm)		9.247	8.236	10.2	0.268	
Estrogen level		39.20	42.33	31.52	0.206	
ER expression		70.31	67.87	77.50	0.686	
Clinical staging	Grade 1	4	4	0	0.229	5.619
	Grade 2	16	26	1		
	Grade 3	16	11	3		
Pain level	light	15	31	0	0.000	16.882
	mild	21	8	4		
Hematomas	no	18	7	3	0.040	11.286
	yes	18	32	1		
Nipple necrosis	no	23	37	4	0.001	12.670
	yes	13	2	0		
Nipple paresthesias	no	10	25	2	0.007	9.937
	yes	26	14	2		
Effusions	no	6	22	1	0.002	12.976
	yes	30	17	3		

### Correlation between surgical recurrence and clinical indicators

Among the 79 surgical patients, the correlations between surgical recurrence and clinical indicators were analyzed, as shown in Table 4. Twenty-six patients relapsed after surgery; 53 patients did not relapse. The two groups of patients were comparable in age and clinical grade (P > 0.05); however, the two groups differed significantly with respect to the estrogen level (P < 0.01) and the proportion of patients undergoing each type of surgery (P = 0.012). The difference in the glandular tissue thickness based on the color Doppler ultrasound also reached statistical significance between the two groups (P = 0.050). Thus, we concluded that patients with a greater glandular tissue thickness and a higher estrogen level had a higher risk of recurrence. The recurrence rate was higher in those undergoing a minimally invasive procedure.

**Table 4:** Correlation between surgical recurrence and clinical indicators

Indicators		Recurrence	No recurrence	P-value
Number		26	53	
Age(year)		29.55	27.42	0.346
Glandular tissue thickness(mm)		10.23	8.09	0.050
Estrogen level		48.41	37.36	0.009
ER expression		70.96	69.74	0.595
Clinical Staging	Grade 1	1	7	
	Grade 2	11	30	0.092
	Grade 3	14	16	
	open surgery	6	30	
Surgical types	minimally invasive rotary cutting surgery	19	20	0.012
	mammoscopic-assisted glandular resection	1	3	

### Correlations between ER and different indicators

**Fig. 1:**
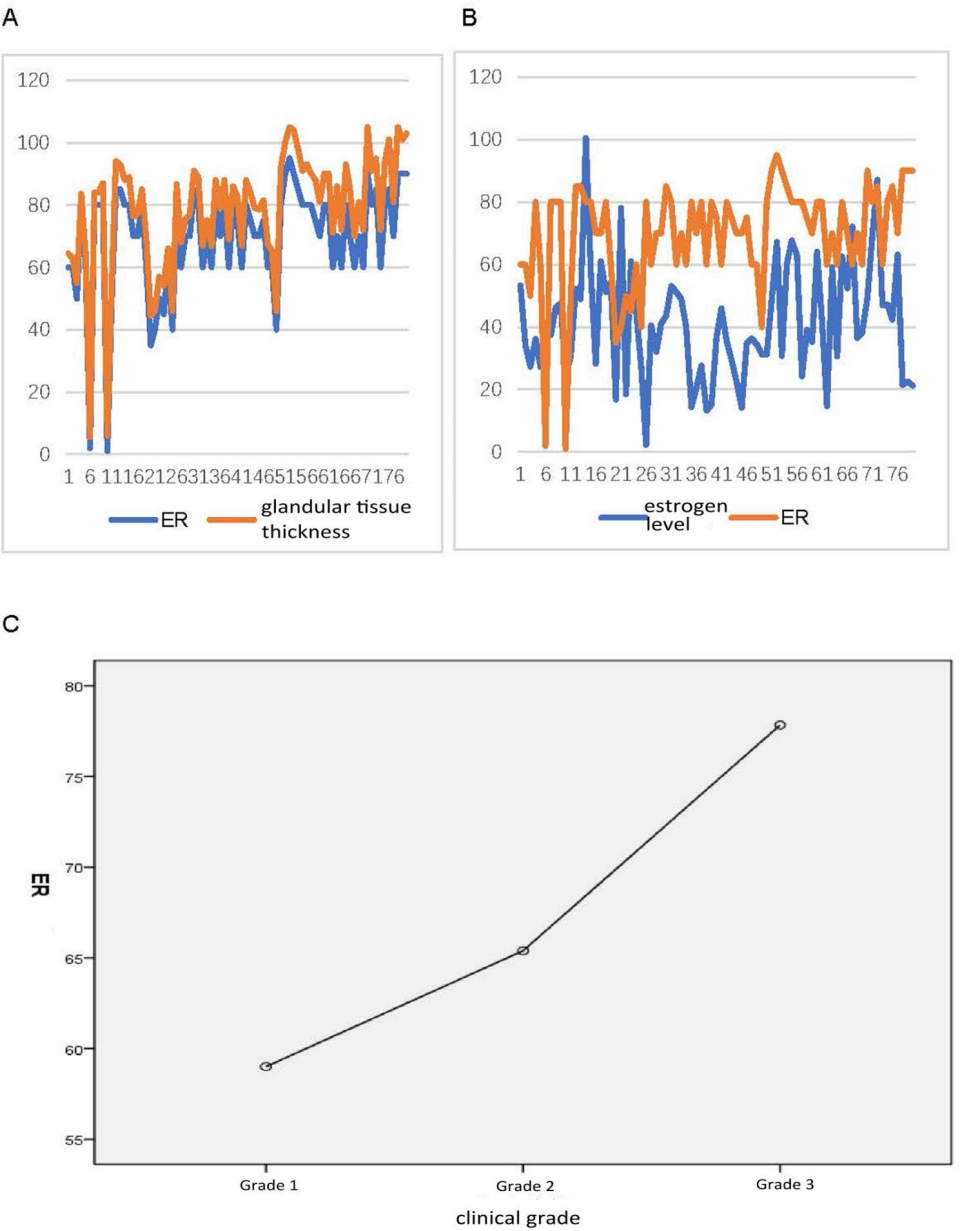
The correlation of ER expression with glandular tissue thickness (**A**), estrogen level (**B**), and clinical grade (**C**)

ER expression was not significantly correlated with glandular tissue thickness, clinical grade, or estrogen level (Fig. 1).

### Results of multivariate analysis

Logistic regression analysis was performed to determine the impact of each clinical indicator on efficacy. The estrogen level and type of treatment had an influence on efficacy (P > 0.05). An elevated estrogen level was a factor leading to TAM failure (P < 0.01; Table 5). Among the surgical patients, logistic regression analysis was performed to assess the correlation between each clinical indicator and surgical recurrence. The glandular tissue thickness, estrogen level, and type of surgery performed were risk factors leading to postoperative recurrence (all P < 0.05; Table 6).

**Table 5:** Logistic regression analysis was performed to determine the impact of each clinical indicator on efficacy

Indicators	P-value	OR	95% interval
Age(year)	0.683	0.995	0.969–1.021
Glandular tissue thickness(mm)	0.549	1.072	0.854–1.346
Clinical Staging	0.896	1.084	0.323–3.633
Estrogen level	0.007	1.035	1.009–1.061
Grouping	0.000	0.097	0.043–0.216

**Table 6:** Logistic regression analysis was performed to determine the impact of each clinical indicator on recurrence in surgery group

Indicators	P-value	OR	95% interval
Age(year)	0.510	0.977	0.911–1.048
Glandular tissue thickness(mm)	0.019	1.613	1.082–2.459
Clinical Staging	0.280	0.341	0.048–2.406
Estrogen level	0.020	1.074	1.006–1.075
ER expression	0.257	0.978	0.941–1.016
Surgery types	0.014	3.740	1.307–10.697

## Discussion

Gynecomastia is a breast disease that specifically involves males. The incidence of gynecomastia has been on the rise due to lifestyle changes and the increasing prevalence of obesity and diabetes [18, 19]. Androgen abuse, use of drugs promoting male breast development, and exposure to environmental endocrine disruptors are considered reasons for the increasing prevalence of gynecomastia [19]. Gynecomastia may also be a clinical manifestation of male breast cancer [20, 21]. This fact highlights the need for clinical management of gynecomastia.

The male hormones most often studied concerning gynecomastia include estrogens, progestogens, androgens, luteinizing hormone, and growth hormone. Thyroid hormones have also been the focus in some studies. According to most studies, abnormal estrogen levels can result in male breast development for the following reasons [1–5, 21]: (1) endocrine disorders and estrogen-androgen imbalance; (2) abnormal estrogen metabolism; (3) elevation of the estrogen level induced by exogenous drug uptake, such as drugs for urinary tract diseases and prohibited substances; (4) hypersensitivity to ERs, leading to mammary gland hyperplasia; and (5) excessive androgen consumption or abnormal endocrine function. Estrogen receptor (ER), a member of the nuclear receptor superfamily, mediates various effects of estrogen. ER is also closely related to the differentiation and proliferation of mammary epithelial cells and tumorigenesis [5, 21, 22]. TAM is an ER antagonist that competitively binds to ER in breasts. TAM reduces a series of effects induced by estrogen by inhibiting estrogen binding to the ER [6, 7, 10–12]. These studies have also demonstrated that TAM can be used as an effective agent in gynecomastia [6, 7, 10–12]. It has also been shown that males are tolerant of TAM with few adverse events reported [23]. TAM works by binding to the ER, which partially explains the variability of responsiveness to TAM [5]. Among patients receiving TAM treatment in the present study, those achieving a more substantial decrease in glandular tissue thickness had a higher estrogen level. By contrast, patients with similar glandular tissue thicknesses also had comparable estrogen levels. The above differences were statistically significant. Multivariate analysis also showed that the efficacy of TAM treatment was significantly correlated with the estrogen level. Moreover, the efficacy of TAM treatment was also significantly correlated with clinical grade. The patients receiving TAM treatment were older in the present study. Thus, we recommend TAM for elderly patients who cannot tolerate surgery, have a low estrogen level, and clinical grade 1–2.

Gynecomastia mainly presents with breast enlargement and pain, which negatively impacts the quality of life. In addition, patients with gynecomastia are prone to anxiety and other psychological problems. Because of psychological and cosmetic considerations, many patients choose to undergo surgical treatment [1, 4, 9]. This is especially the case for patients with clinical grade 2–3. The surgery usually involves a resection of the hyperplastic mammary glands to improve the appearance of the enlarged breasts and dispel patients’ worries and concern [12, 13]. Conventional open surgery can achieve complete resection of hyperplastic mammary glands, although causing conspicuous scars in the breasts, and prolonging the hospital stay and time to healing. Many patients have severe pain after conventional open surgery along with other adverse reactions, including edema, bruises, postoperative effusions, or nipple necrosis [24, 25]. The minimally invasive procedure is less traumatic and shortens the hospital stay and time to healing. The patients are more satisfied with a minimally invasive procedure, although the problems of postoperative hematomas, effusions, gland residue, and postoperative recurrence still exist [24, 25]. Mammoscopic-assisted glandular resection is a safe and feasible procedure for treating gynecomastia, causing few adverse reactions except for mild pain and edema. Mammoscopic-assisted glandular resection is often described as having a long learning curve and highly-skilled surgeons, which pose obstacles to the wider clinical application of this procedure [26, 27]. The present study included an insufficient number of patients undergoing mammoscopic-assisted glandular resection for an efficacy evaluation of the procedure. In the present study, the patients receiving an open surgery were older in age, had a prolonged stay in hospital, and suffered from moderate pain after surgery compared with the other two surgical procedures. Those patients undergoing open surgery had more frequent adverse reactions, including nipple necrosis and effusions, the findings of which are in agreement with previous reports [24, 25]. The minimally invasive rotary cutting surgery resulted in a shorter length of hospital stay and less severe pain compared with the other two procedures. Hematoma was a common postoperative complication. However, the minimally invasive rotary cutting surgery was associated with a higher risk of surgical recurrence, which was consistent with the previous reports [24, 25]. Therefore, we recommend open surgery for patients with more redundant skin and clinical grade 2–3; however, the minimally invasive rotary cutting technique is preferred for patients with clinical grade 1–2 and a smaller scope of glandular hyperplasia. The surgical patients were further divided into recurrence and non-recurrence subgroups. The two subgroups were not significantly different in age and clinical grade; however, the glandular tissue thickness was smaller in the non-recurrence group than the recurrence group. The estrogen level was also considerably lower in the non-recurrence group than the recurrence group. Given the above findings, we believe that patients with a greater glandular tissue thickness and a higher estrogen level are at a higher risk of recurrence. In addition, recurrence was more common after the minimally invasive procedure. Therefore, we suggest that the type of surgery should be chosen based on the clinical grade, glandular tissue thickness, estrogen level, and the patient’s willingness to undergo treatment.

Thus far, the minimally invasive rotary cutting technique is more widely accepted among patients. Young patients usually have a stricter requirement for breast appearance, a higher acceptance of new technologies, and achieve more rapid postoperative healing [24, 28, 29]. In the present study, such patients tended to have a higher estrogen level and less significant breast enlargement. Specifically, these patients had hyperplastic glands in the areola and no redundant skin, and the size of resection was small. The minimally invasive procedure is guided by B-mode ultrasound, which offers an accurate localization of the hyperplastic glands. The area of resection with the minimally invasive procedure is limited, which increases the risk of residual glands [24, 25, 28, 29]. Postoperative estrogen level monitoring is necessary for these patients. Oral TAM should be prescribed for ER-positive patients with a high estrogen level and preoperative glandular thickening to prevent postoperative recurrence.

### Limitations

In the present study, we excluded teenage males < 18 years of age for the following reasons: oral TAM may cause greater side effects among adolescents; and transient mammary gland development caused by hormonal changes is reversible to a certain degree. The exclusion of adolescent males, however, might result in biases. Moreover, the present study was only conducted in a single center. Multi-center studies with a larger sample size with long-term follow up are warranted.

## Conclusion

Our study results show that TAM and surgery treatments can effectively treat gynecomastia, but an individualized treatment regimen is recommended for these patients based on their clinical features. TAM is more beneficial than TAM for patients who cannot tolerate surgery, have a low estrogen level, and are clinical grade 1–2. Surgery treatment is better than TAM for patients of clinical grade 3. Different surgery options may lead to different complications. The patients with a greater glandular tissue thickness and a higher estrogen level were shown to have a higher risk of recurrence.

## Data Availability

The datasets analysed during the current study are available from the corresponding author on reasonable request.

## List of abbreviations

TAM: Tamoxifen
hGH: Human growth hormone
hCG: Human chorionic gonadotrophin
ER: Estrogen receptor
